# Meeting the challenges of invasive alien species

**DOI:** 10.1093/nsr/nwae017

**Published:** 2024-01-11

**Authors:** Weijie Zhao

## Abstract

Here we report the Assessment Report on Invasive Alien Species and their Control released by IPBES, and the status of IAS in China.

The Xinjiang Uygur Autonomous Region of China is famous for its tomato production. Abundant sunshine, large day–night temperature difference and the abundant irrigation by snowmelt from the Tianshan Mountains produce high-quality tomatoes with excellent taste and rich nutrition. However, the Xinjiang tomato has been threatened since 2017 by the tomato leaf miner (*Tuta absoluta*)—a moth known to be a pest that is highly destructive to the world tomato industry. It originated in Peru and has now spread from Xinjiang and Yunnan to ∼20 provinces in China. On the global scale, it has invaded >100 countries in Europe, Africa, Asia and North America. On tomatoes alone, it causes a direct economic loss of more than ∼$5.7 billion per year.

Prof. Wanxue Liu (刘万学) and his colleagues from the Institute of Plant Protection of the Chinese Academy of Agriculture Sciences have worked on the identification, monitoring, prevention and control of the leaf miners (Fig. 1). Their integrated approaches have achieved some success, but long-term effort is still needed to sustainably control these pests in China.

**Figure 1.**
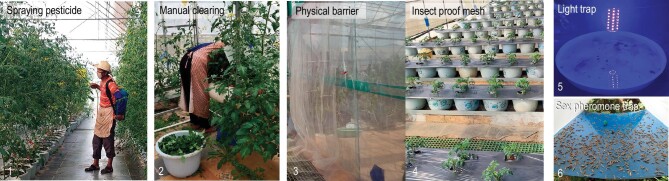
Integrated approaches to control tomato leaf miners in China. *(Courtesy of Prof. Wanxue Liu)*

Invasive alien species (IAS) are one of the five major causes of biodiversity loss and have brought severe harm to food production, biological and ecological security, as well as human health. The IAS plays a key role in 60% of global species extinctions and brought about an annual economic cost of $423 billion globally in 2019, according to the latest version of the *Assessment Report on Invasive Alien Species and Their Control* [1], which was approved by representatives of 143 member states of the Intergovernmental Science-Policy Platform on Biodiversity and Ecosystem Services (IPBES) on 4 September 2023 in Bonn, Germany. This IPBES Assessment Report was produced through the collective efforts of 86 nominated experts from 49 countries and ∼200 contributing authors over a period of 4.5 years.

Prof. Ning Wu (吴宁), the director general of Chengdu Institute of Biology, Chinese Academy of Sciences, is a member of the IPBES Multidisciplinary Expert Panel. He noted: ‘The *Assessment* cited many papers published by Chinese researchers about IAS, thus providing an important platform for international society to learn the Chinese experience in controlling and managing IAS.’ Some Chinese experts also participated in the manuscript review and provided constructive suggestions.

As the most comprehensive IAS assessment ever, the *Assessment* has collected international data as much as possible, including official publications, peer-reviewed articles and evidence from indigenous and local information. ‘However, due to the data-unbalance among regions and countries, for some countries there are more cases or evidence cited in this report than others,’ said Wu. For example, for the Asia-Pacific region, most of the data and publications are from China, Japan, Korea, India, Australia and New Zealand. For other countries, the data are rather limited, showing many gaps in data for the Asia-Pacific region. Even so, this report has updated our knowledge about IAS in the world, including the status, trend, direct and indirect drivers, as well as potential scenarios in the future.

## IAS THROUGHOUT THE WORLD

**Figure 2.**
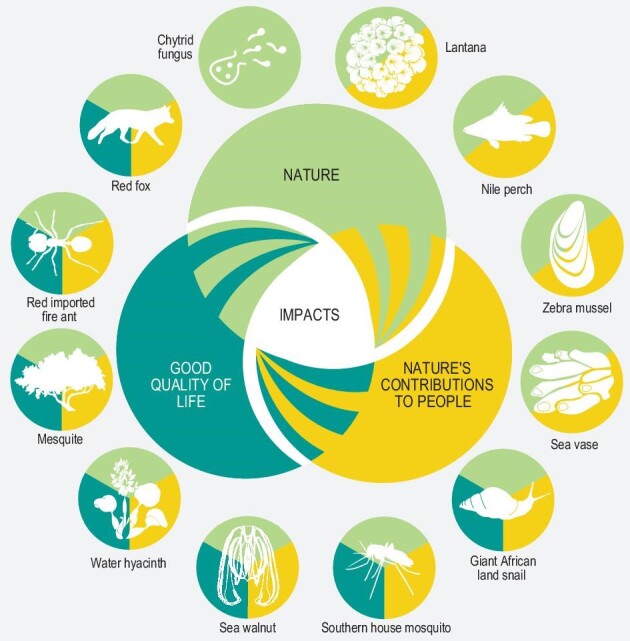
Examples of invasive alien species with a negative impact on nature (green), nature's contributions to people (yellow) and/or good quality of life (teal). Reproduced from the open-access IPBES *Assessment* [1].

Species introduced to new regions through human activities are termed alien species and a subset of them becomes IAS, which are, as Wu explained, those that ‘have established and spread with negative impacts on biodiversity, local ecosystems and species’. According to the *Assessment* (Fig. 2), there are >37 000 established alien species around the world, among which >3500 are invasive, including 141 microbes, 1061 plants and 2313 animals (1852 invertebrates and 461 vertebrates). In China, according to the *2020 Bulletin of Ecological-Environmental Situation* issued by the China's Ministry of Ecological Environment, there are >660 IAS, including ∼370 plants and ∼220 animals, among which 120 species have a serious impact on agricultural production and 71 species have impacted or will potentially have an impact on ecosystems.

The first nationwide IAS general survey is currently ongoing in China. The survey is led by the Ministry of Agriculture and Rural Affairs in collaboration with six other ministries and will take ∼3 years [2]. It aims to identify the total number, distribution and harm of IAS in China and to conduct in-depth investigation into the prevention and control of the key IAS.

‘Many invasive alien species have been intentionally introduced for their perceived benefits without enough consideration or knowledge of their negative impacts,’ said Wu. For example, the water hyacinth (*Pontederia crassipes*), which is the world's most widespread IAS, was introduced into China in the 1950s as a pig feed. But it was soon discarded by farmers and escaped into the wild. Its extensive growth blocks waterways and impairs shipping, irrigation and aquaculture. It also blocks the sunlight needed for underwater organisms, leading to severe water pollution and destroying the ecosystem.

In many other cases, IAS were unintentionally introduced through contaminants of traded goods or stowaways in shipments. The red imported fire ant (*Solenopsis invicta*) was originally found in local areas of South America and has invaded ∼30 countries throughout South, Central and North America, Oceania and Asia. It was first found in China in 2003 in Taiwan and in 2004 in Guangdong. Now, it has rapidly spread into more than a dozen provinces across South China. These ants are highly aggressive. They have poisonous needles and are ready to attack animals and humans when disturbed, causing pain, swelling, fever, temporary blindness, and even shock and death in severe cases. Feeding on buds, stems, flowers and seeds, they may also significantly reduce the yield and quality of crops.

Notably, some alien species only begin to spread and become invasive long after the first introduction, meaning that the magnitude of the future impact of IAS may be underestimated based on current observations.

Under a ‘business-as-usual’ scenario, which assumes that trends of drivers will continue as observed in the past, the *Assessment* predicts that the total number of alien species globally by 2050 will be about one-third higher than that in 2005. ‘But business-as-usual is actually unlikely,’ said one of the three co-chairs of the *Assessment*, Professor Helen Roy, in the IPBES news release [3]: ‘With so many major drivers of change predicted to worsen, it is expected that the increase of invasive alien species and their negative impacts, are likely to be significantly greater. The accelerating global economy, intensified and expanded land- and sea-use change, as well as demographic changes are likely to lead to increases in invasive alien species worldwide. Climate change will make the situation even worse.’

## MANAGEMENT OF IAS

IAS management is an urgent issue for the world but the current national policies have been inadequate. According to the *Assessment*, although 80% of countries have targets for the management of biological invasions, 83% do not have national legislation or regulations directed specifically toward the prevention and control of IAS. China is among the 17% that have such legislation. On 15 April 2021, the Biosafety Law of the People's Republic of China came into effect. On 17 June 2022, the Regulation of Management of Invasive Alien Species was issued by four Chinese ministries.

Prevention and preparedness are the most cost-effective options for IAS management. ‘It can be achieved through pathway management, including strictly enforced import controls, pre-border, border and post-border biosecurity, and measures to address escape from confinement,’ said Wu. For example, Chengdu, as an important transport hub in west China linking central Asia and south Asia through the newly constructed railways, biosecurity control of international freightage has been developed.

Eradication, containment and control are also effective options when the IAS has already caused destruction. Physical, chemical, biological and integrated approaches could be used. Liu pointed out four major difficulties: first, some species can spread rapidly through diverse pathways; second, the outbreaks of IAS often come suddenly without obvious warnings; third, the IAS populations easily recover after eradication; and finally, more technologies, human power and financial support are needed.

One example is China's attempt to manage saltwater cordgrass (*Spartina alterniflora*) [4]. Saltwater cordgrass can tolerate salty sea water and grow on tidal beaches, and it was introduced from the USA to China in the late 1970s to protect the shorelines from typhoons. However, the expected ‘protector of coastlines’ became invasive and had covered nearly half of China's salt marshes by 2019.

In late 2012, the State Forestry Administration (currently known as the National Forestry and Grassland Administration) and the Shanghai government launched a project to tackle saltwater cordgrass in the Eastern Beach of Chongming island. The eradication approach include two steps: first cut down the grass and then submerge the beach in water for ≥6 months to completely kill the roots. Completed in 2018, the project was a great success—95% of cordgrass was eliminated, and the local plants and birds began to come back.

However, this project was very expensive, costing 1.16 billion yuan (∼$159 million), and is hard to replicate in other provinces. In 2022, five ministries issued the Special Action Plan for the Prevention and Control of *Spartina alterniflora* (2022–2025), aiming to eliminate >90% of the cordgrass in all provinces by 2025.

Wu noted that, for saltwater cordgrass and many other IAS: ‘Due to the varied and complicated natural and socio-economic conditions of different regions in China, context-specific application of integrated approaches should be very important. More than one containment or control option should be used.’

China is the one of the countries that are most severely affected by IAS, but it seems that IAS still needs more Chinese media coverage. There are obvious reasons for this: the study and attention of IAS began late in China and public dissemination of the knowledge has lagged behind.

Fortunately, alongside the related research and management efforts, science popularization activities have become more active in recent years. Public activities such as online and offline special lectures, open-day activities of research institutions and fishing competitions of invasive fish species have raised the public awareness of IAS, especially among children and teenagers, and promoted societal participation in IAS prevention and control.

Inger Andersen, the executive director of the United Nations Environment Programme, appealed in a recent IPBES news release: ‘I ask all decision-makers to use this report's recommendations as a basis to act on this growing threat to biodiversity and human well-being—and make a real contribution to achieving the Kunming-Montreal Global Biodiversity Framework by 2030.’ The Framework mentioned here was issued in December 2022 and its Target 6 aims to tackle the impacts of IAS on biodiversity and ecosystem services, and to reduce the rate of introduction and establishment of IAS by ≥50% by 2030.
